# Cholesterol granuloma presenting as a mass obstructing the external ear canal

**DOI:** 10.1186/1472-6815-10-4

**Published:** 2010-04-05

**Authors:** Vasilios Nikolaidis, Hariklia Malliari, Dimosthenis Psifidis, Spyridon Metaxas

**Affiliations:** 1 2nd Department of Otolaryngology, Aristotle University., Papageorgiou G.H., Thessaloniki, Greece

## Abstract

**Background:**

Cholesterol granuloma (CG) may involve the middle ear, the mastoid bone and the petrous apex. However, CG presenting as a mass obstructing the external ear canal (EEC) is relatively rare and it can be a diagnostic challenge.

**Case Presentation:**

We report a case of a CG occupying the mastoid antrum and presenting as a mass into the EEC. Temporal bone computerized tomography showed a soft tissue mass which eroded the posterior-superior bony wall of the EEC. On magnetic resonance imaging, the mass revealed a high signal on both T1 and T2-weighted images. The CG was removed by a mastoidectomy procedure and the histopathologic report confirmed the diagnosis of CG. A type III tympanoplasty was performed.

**Conclusions:**

The postoperative course was uneventful.

## Background

Cholesterol granuloma (CG), or cholesterol cyst, is a clinical entity that was first reported by Manasse in 1894 [1]. It appears as an expansible benign mass that contains brownish-yellow debris with cholesterol crystals and is characterized by slow growth. The lesion can be found in any part of the body where deposition of cholesterol crystals may occur though the temporal bone and specifically the petrous apex is the most common site [2,3]. This condition is more often recognized nowadays than in the past but this might reflects better diagnosis rather than an increase of incidence. However, CG presenting as a mass obstructing the external ear canal (EEC) is rare and it can be a diagnostic challenge. We were able to trace only three cases from the international literature [4-6]. We present our experience with a case of CG in the EEC and review the relevant medical literature.

## Case presentation

**Figure 1:**
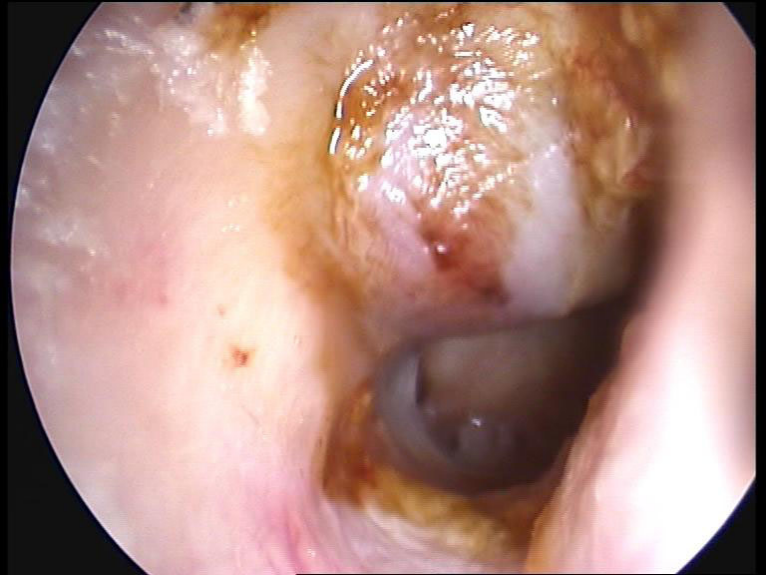
**Cholesterol granuloma occupying the posterior- superior part of the external ear canal**.

A 65 year old male presented to our ENT clinic as an outpatient for a routine yearly check up. He had a history of chronic otitis media with subtotal ear drum perforation in both ears. Left myringoplasty was performed thirty years ago. Otoscopic examination revealed stenosis of the right EEC due to the presence of a mass occupying the superior-posterior part of the canal, which prevented visualization of almost half of the perforated tympanic membrane (Figure 1). The middle ear had no signs of infection. Audiometry showed a moderate conductive hearing loss. The patient didn't detect any changes in his ear condition.

**Figure 2:**
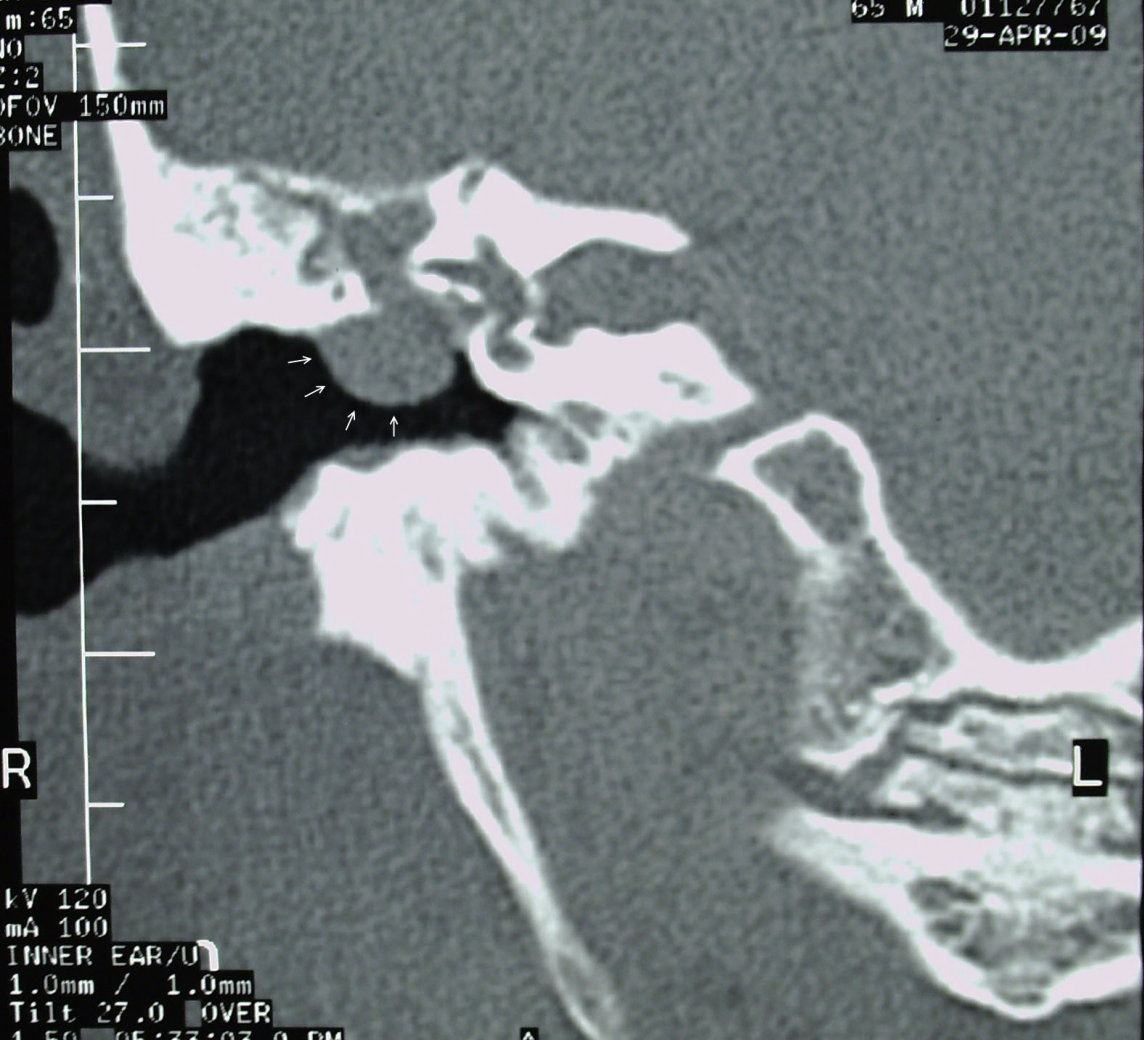
**Temporal bone CT (coronal) showing a soft tissue mass occupying the mastoid antrum and part of the external ear canal**. Arrows indicate the mass expanding into the external ear canal.

**Figure 3:**
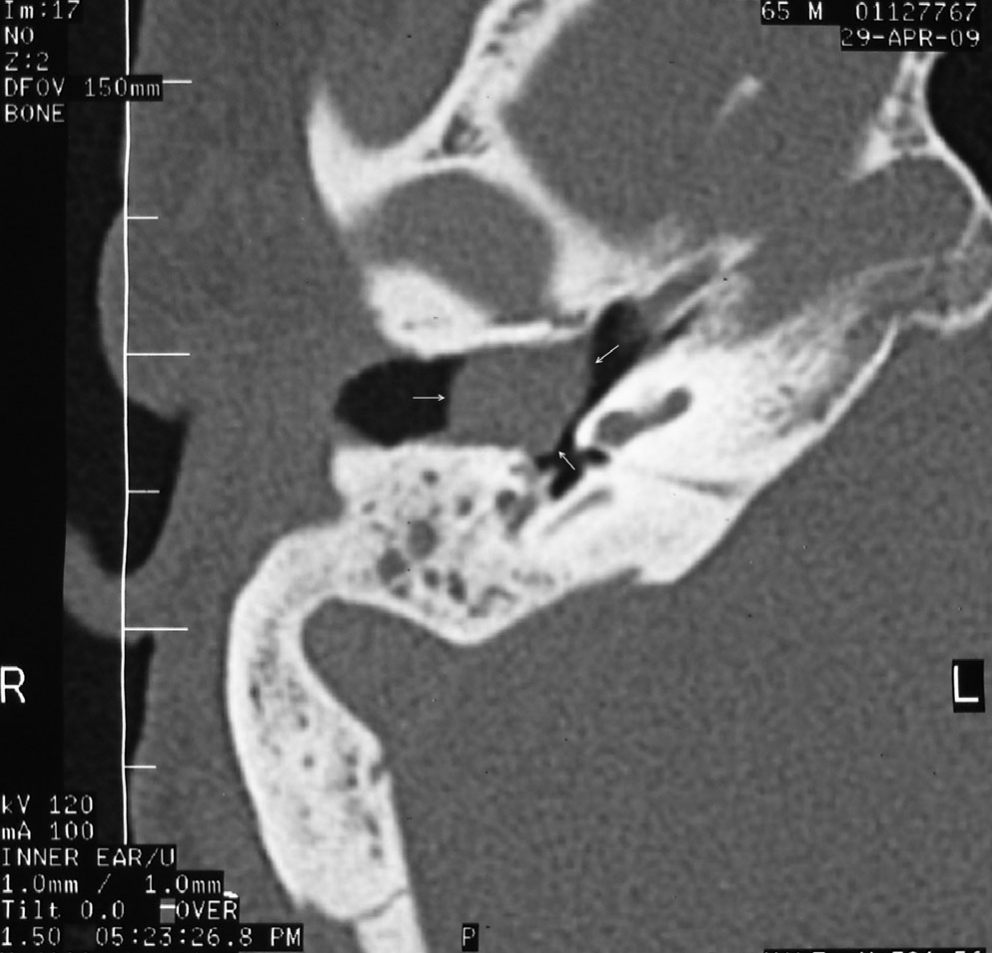
**Temporal bone CT (axial) showing a soft tissue mass occupying part of the external ear canal**. Arrows indicate the mass into the external ear canal.

**Figure 4:**
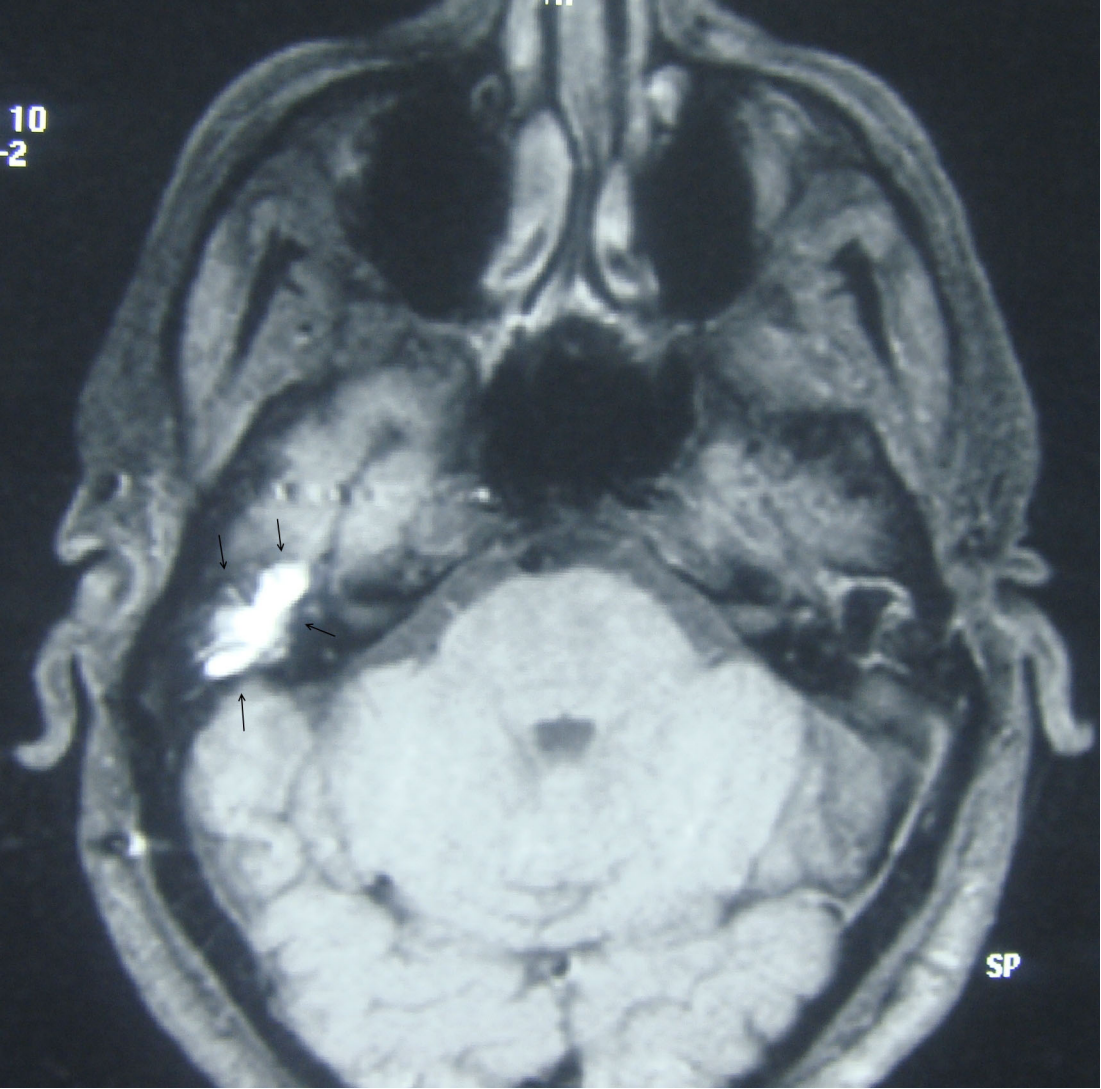
**T1-weighted temporal bone MRI showing a homogenous mass with increased signal intensity relatively to the brain**. Arrows indicate the mass.

**Figure 5:**
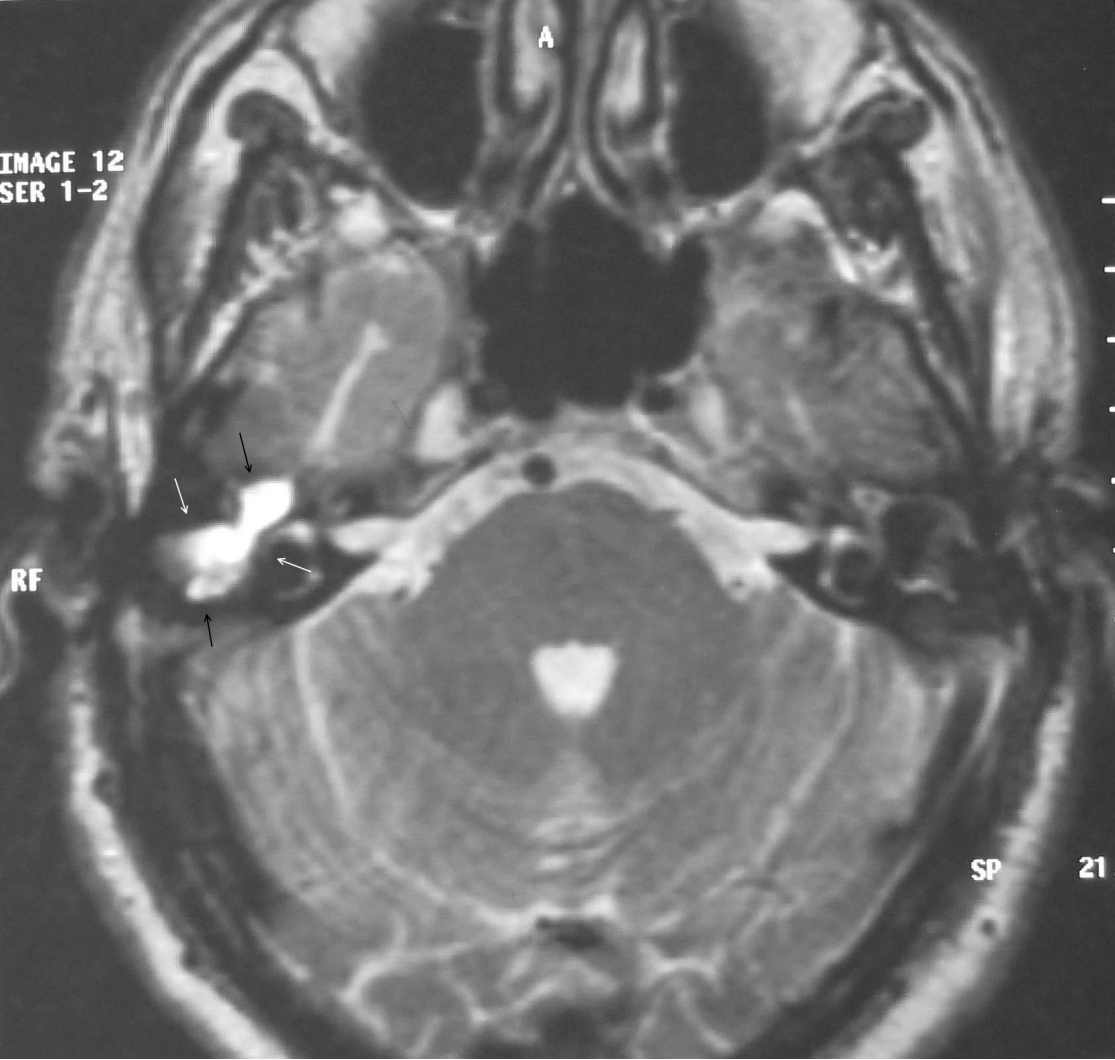
**T2-weighted temporal bone MRI showing a homogenous mass with increased signal intensity relatively to the brain**. Arrows indicate the mass.

Temporal bone CT (Figure 2 and 3) showed a soft tissue mass in the right EEC. The posterior-superior bony wall of the EEC was eroded; the mass expanded into the mastoid antrum and was separated from the middle cranial fossa by a thin bony plate. Erosion of the ossicles of the right ear was also present. At MRI the mass appeared homogeneous with increased signal intensity relatively to the brain on both T1- and T2- weighted images (Figure 4 and 5). Inner ear formations were normal in both ears and pneumatization of both temporal bones was poor. The diagnosis of CG was supposed.

Blood work was normal. The serum cholesterol levels of our patient were within normal limits. The cyst was drained and the brownish semi-liquid material was cultivated; no microorganisms where found. Cholesterol was found to be present in the effusion (274 mg/dL).

Through a postauricular approach the patient underwent mastoidectomy. The mastoid antrum was entered and the cyst was excised along its direction to the EEC. The posterior canal wall was reconstructed with a piece of auricular cartilage. Then a type III tympanoplasty was performed. The postoperative course was uneventful. The histopathologic report confirmed the diagnosis of CG. The cyst had a fibrous lining and contained cholesterol crystals, haemosidirin and fibrin which were surrounded by foreign-body giant cells.

## Conclusions

The first theory about the pathogenesis of the cholesterol cyst was proposed by Friedman in 1974 [7], according to which local hemorrhage and the catabolism of hemoglobin produces cholesterol crystals and iron. Sade [8] suggested an alternative theory in 1979, emphasizing the role of low ventilation and hypoxia implying that cholesterol derives from disintegrated tissue and that iron is associated with lactoferrin. More specific theories have also been formulated. In 1964 McNaughton [3] linked the herpes simplex virus with CG of the middle ear. The latest theory concerning petrous apex CG is the exposed marrow theory by Jackler and Cho [9] in 2003, who suggested that an aggressive and abnormal pneumatization of the mastoid cells in young adulthood results in exposed bone marrow which can lead to future hemorrhage. Summarizing, there are three major factors regarded as responsible for the formation of a CG, local hemorrhage which can occur during an inflammatory process, obstruction of ventilation and poor drainage of the cavities of the middle ear [10].

Microscopically, the cyst presents cholesterol clefts (the cholesterol dissolves during tissue processing) that are surrounded by histiocytes and foreign-body giant cells [11]. In fact the cholesterol crystals stimulate the accumulation of giant cells which are responsible for the tissue reaction [12].

The clinical presentation of the CG is variable since it is encountered in different locations and dimensions. It could be discovered incidentally, as in our case, or can be presented with hearing loss (conductive when concerning the middle ear or sensoneurinal when located in the petrous apex), tinnitus, vertigo, cranial nerve deficits, headache, facial pain/numbness, diplopia and CSF leak [13,14]. The differential diagnosis depends on the site of the lesion (Table 1).

**Table 1:** Differential diagnosis according to the lesion site.

PETROUS APEX	MIDDLE EAR CAVITY	EXTERNAL MEATUS
Glomus tumor	Cholesteatoma	Osteoma

Meningioma	High jugular bulb	Bony stenosis

Schwannoma	Carotid artery aberrance	Cholesteatoma

Arachnoid cyst	Glomus tumor	Brain hernia

Plasmocytoma	Hemotympanum	

Lymphoma	Brain hernia	

Histiocytosis X		

Epidermoid cysts		

Mucocele		

Carotid aneurism		

Bone and cartilaginous tumors		

Computed tomography scanning and magnetic resonance imaging plays a significant role in the diagnosis of CG. The CG appears as a well-marginated lesion in the CT, isodense with the brain, with no significant contrast enhancement while bone erosion may be present. MRI is helpful in order to make differential diagnosis from other non-contrast-enhanced masses such as cholesteatomas and brain hernias and to conduct a proper preoperative plan. The CG has short T1 and long T2 features and amplified signal intensity in both T1 and T2 weighted images [15].

Regarding the treatment of CG, variable approaches are plausible. The treatment plan depends on the location and size of the lesion. There are reports of conservative therapy such as simple patient follow-up with serial neurological exams and MR imaging [13]. Medical treatment using steroids is also suggested [16]. In more severe cases surgical approach is preferred. Since the cholesterol granuloma lacks epithelial lining often total excision is not possible. It is supported that surgical removal of the cyst, proper drainage and permanent ventilation will ensure that there will be no recurrence [17].

## Consent

Written informed consent was obtained from the patient for publication of this case report and accompanying images. A copy of the written consent is available for review by the Editor-in-Chief of this journal.

## Competing interests

The authors declare that they have no competing interests.

## Authors' contributions

VN study design, involved in drafting the manuscript and revising it critically. HM study design, involved in drafting the manuscript. DP study design, involved in drafting the manuscript. SM study design, involved in drafting the manuscript and revising it critically. All authors read and approved the final manuscript.

## Pre-publication history

The pre-publication history for this paper can be accessed here:

http://www.biomedcentral.com/1472-6815/10/4/prepub
